# Microbial Diversity of the Chinese Tiger Frog (*Hoplobatrachus rugulosus*) on Healthy versus Ulcerated Skin

**DOI:** 10.3390/ani12101241

**Published:** 2022-05-12

**Authors:** Hua-Li Hu, Jia-Meng Chen, Jing-Yi Chen, Rachel Wan Xin Seah, Guo-Hua Ding

**Affiliations:** 1 Laboratory of Amphibian Diversity Investigation, College of Ecology, Lishui University, Lishui 323000, China; huhuali0814@foxmail.com (H.-L.H.); chenjiam077@163.com (J.-M.C.); jinychen1207@outlook.com (J.-Y.C.); 2 Department of Biological Science, National University of Singapore, Singapore 117558, Singapore; rachelseah@nus.edu.sg

**Keywords:** *Hoplobatrachus rugulosus*, skin microbiota, skin ulcer, 16S rRNA

## Abstract

**Simple Summary:**

As amphibians’ skin is highly sensitive to the environment, skin defects such as ulceration may pose a particular threat to them. Our study has found a stark difference in the microbial communities between healthy and ulcerated *Hoplobatrachus rugulosus* skin. The proportion and type of bacteria differed between the two groups, and we suggest that ulceration on the skin may lead to changes in skin microbial communities. The functional pathways of skin microbes may be influenced by ulceration on the skin surface of *H*. *rugulosus*. We also found that *Vogesella* is more abundant in healthy *H*. *rugulosus*, which may be a potential probiotic candidate for the reduction or removal of pathogens.

**Abstract:**

The Chinese tiger frog (*Hoplobatrachus rugulosus*) is extensively farmed in southern China. Due to cramped living conditions, skin diseases are prevalent among unhealthy tiger frogs which thereby affects their welfare. In this study, the differences in microbiota present on healthy versus ulcerated *H. rugulosus* skin were examined using 16S rRNA sequences. Proteobacteria were the dominant phylum on *H. rugulosus* skin, but their abundance was greater on the healthy skin than on the ulcerated skin. Rhodocyclaceae and Comamonadaceae were the most dominant families on the healthy skin, whereas Moraxellaceae was the most dominant family on the ulcerated skin. The abundance of these three families was different between the groups. *Acidovorax* was the most dominant genus on the healthy skin, whereas *Acinetobacter* was the most dominant genus on the ulcerated skin, and its abundance was greater on the ulcerated skin than on the healthy skin. Moreover, the genes related to the Kyoto Encyclopedia of Genes and Genomes pathways of levels 2–3, especially those genes that are involved in cell motility, flagellar assembly, and bacterial chemotaxis in the skin microbiota, were found to be greater on the healthy skin than on the ulcerated skin, indicating that the function of skin microbiota was affected by ulceration. Overall, the composition, abundance, and function of skin microbial communities differed between the healthy and ulcerated *H. rugulosus* skin. Our results may assist in developing measures to combat diseases in *H. rugulosus*.

## 1. Introduction

Amphibians are vertebrates that transition from aquatic to terrestrial habitats and play vital roles in maintaining the balance of various ecosystems. As one of the countries with the most abundant diversity and number of amphibians in the world, China has a long history of utilizing and consuming amphibians [1,2,3,4]. Owing to the increasing demand for amphibians, the numbers required may far exceed wild populations, which may result in a decline of wild amphibian species [5]. Therefore, artificial breeding of amphibians helps meet consumer demand while reducing the pressure on wild amphibian populations [2,6]. However, the development of amphibian farming is accompanied by the incidence and spread of diseases that could be caused by poor farming practices such as cramped conditions and inadequate nutrition [7]. These diseases are usually caused by bacteria [8,9], fungi [10,11,12,13], viruses [14], parasites, and other pathogens, all of which can result in high mortality and incur huge economic losses to the breeding industry [15].

The amphibian skin serves as a vital organ for maximizing air exchange and hosts a variety of microbial communities that defend the organism against pathogenic invasion [7,16,17]. Furthermore, the interactions among the host, symbiotic microorganisms, and pathogens generally affect the outcome of diseases [18] and often heighten the individual’s immunity against pathogens [19]. As the skin microbiome may influence an amphibian’s susceptibility to a disease, the changes in the diversity of skin-related microbiota can lead to a greater risk of infection [20,21,22]. A common pathogenic symptom of skin disease is ulceration [7], which is caused by various physical or chemical factors. Additionally, alterations in the skin microbiome may lead to an increase in pathogenic bacteria and subsequent disease, for example, chytridiomycosis. This disease, caused by a highly contagious fungus (*Batrachochytrium dendrobatidis*, [*Bd*]), may be the main reason for the rapid decline and extinction of several amphibian species worldwide [23,24,25]. Researchers have extensively studied both pathogenic and beneficial bacteria of the amphibian skin microbiome [26,27,28]. However, the correlation between amphibian skin diseases and microbial diversity still remains unclear. Moreover, a study of the microbiome diversity of amphibian skin will contribute to our understanding of the role of the skin microbiome composition in protecting and maintaining amphibian health.

The Chinese tiger frog (*Hoplobatrachus rugulosus*) is one of the most economically important amphibian species in China and is consequently being raised in captivity [4]. To meet the high market demand, the development of *H. rugulosus* farming in China has progressed quickly. However, this comes at a cost of frequent outbreaks of diseases among captive populations due to poor living conditions [29]. Diseases such as chytridiomycosis can spread rapidly within the population and adversely affect the productivity of *H. rugulosus* farms.

The 16S rRNA sequence is considered to be the “gold standard” for studies on microbial identification and classification as it contains key information for determining evolutionary relationships between species [30]. Ever since high-throughput sequence-based methods have come into full effect, studies on microorganisms in relation to plants and animals have been increasing as these advanced methods now allow for greater documentation of the structure and composition of a large number of complex microorganisms. In this study, the microbiota from healthy and ulcerated *H. rugulosus* skin was analyzed via 16S rRNA sequencing. The composition, abundance, and function of microbial communities were then compared and analyzed to explore the differences and to better understand the changes in the microbial diversity between healthy and ulcerated skin.

## 2. Materials and Methods

### 2.1. Sample Collection

Eight adult male *H. rugulosus* were randomly selected, four with observably healthy skin and four with ulcerated skin from the froggery at Lishui University. Based on clinical diagnosis, frogs with the presence of local ulceration on the body were considered to have ulcerated skin. Only the dorsal area of the individuals with ulceration of diameter approximately 1–1.5 cm or without ulceration were chosen as the sampling site in this study to reduce experimental errors. Each frog was washed twice with sterile distilled water to reduce transient surface bacteria followed by swabbing of the ulcerated dorsal area of *H. rugulosus* individuals 10 times, yielding one sample per frog. The samples were collected in the same manner for *H. rugulosus* individuals with healthy skin. The swabs were subsequently placed into a sterile bottle. The healthy skin samples were numbered HR1601N, HR1603N, HR1604N, and HR1605N, and they formed the healthy group (HG). The ulcerated skin samples were numbered HR1606U, HR1607U, HR1609U, and HR1610U, and they formed the ulcerated group (UG). The samples were frozen in liquid nitrogen and then stored at −80 °C.

### 2.2. DNA Extraction, Amplification, and Sequencing

DNA was then extracted using the HiPure Stool DNA Kit (Magen, Guangzhou, China), and their quantity and quality were determined using the Qubit^®^ 2.0 Fluorometer (Life Technologies, Carlsbad, CA, USA) and by agarose gel electrophoresis. Subsequently, the V4 region of the bacterial 16S rRNA gene was amplified in 50 μL reaction mixtures using 2× Taq master Mix (TransGen Biotech Co., Ltd., Beijing, China) with 5 μM each of the primers *515F* and *806R*.

According to the operation manual (Gene Denovo Biotechnology Co., Ltd., Guangzhou, China), the thermal cycling conditions were as follows: initial denaturation at 95 °C for 2 min followed by 27 cycles at 98 °C for 10 s, 62 °C for 30 s, and 62 °C for 30 s, and a final extension at 68 °C for 10 min. The products were recovered and quantified with a QuantiFluor™ fluorometer (Promega, Madison, WI, USA). The PCR amplicons were sequenced on the Illumina HiSeq2500 platform (Novogene Bioinformatics Technology Co. Ltd., Tianjin, China). The raw data were deposited in the Sequence Read Archive database of NCBI (BioProject: PRJNA766330).

### 2.3. Data Analysis

FASTP v0.18.0 was used to filter the clean reads by deleting low-quality sequences and primer sequences, and the paired-end reads were merged using FLSAH 1.2.119 [31]. Chimeric reads were identified and removed using the UCHIME algorithm to obtain the clean tags [32]. Operational taxonomic units (OTUs) at the threshold of 97% were clustered using UPARSE [33]. After obtaining the OTUs, relevant software was used to analyze valid sequencing data of skin microorganisms in the HG and UG, respectively. The representative sequences by organisms were classified using a naive Bayesian model with RDP classifier 2.2 and the SILVA Database (https://www.arb-silva.de/; accessed on 7 May 2019), at confidence thresholds ranging between 0.8 and 1 [34,35]. Krona 2.6 was used to visualize the abundance of each taxon [36]. Linear discriminant analysis (LDA) effect size (LEfSe) [37] was used to determine the dominant OTUs (LDA > 4) at different bacterial taxonomic levels from phylum to genus, between healthy and ulcerated skin. Mothur 1.30.1 [38] was used to calculate the alpha diversity indexes, including Shannon index, Simpson index, Chao1 richness, the abundance-based coverage estimator (ACE), and Good’s coverage index. R 3.6 [39] was used to visualize rarefaction curves, including the Shannon index curve and rank–abundance curve. Student’s *t*-test was conducted to compare the difference in alpha diversity indexes between healthy and ulcerated skin. For beta diversity analysis, principal coordinate analysis (PCoA), non-metric multidimensional scaling (NMDS), unweighted pair-group method with arithmetic means (UPGMA), and analysis of similarities (ANOSIM) were performed using vegan package [40] in R 3.6. 

To further our understanding of the functional differences between microbiota present on healthy and ulcerated *H. rugulosus* skin, functional predictions of all OTUs of the skin microbiome were performed using PICRUSt [41] on the Kyoto Encyclopedia of Genes and Genomes (KEGG) database (https://www.genome.jp/kegg/; accessed on 10 May 2019) [42]. These genes were assigned to KEGG pathways and the relative abundance in each group was calculated. We used Welch’s *t*-test to compare the abundance of genes related to functional pathways of levels 1 to 3 between healthy and ulcerated skin. All values are presented as mean ± SD. The differences were considered significant at *p* < 0.05.

## 3. Results

### 3.1. Sequencing Data

The average number of effective sequences of samples was 67,726; OTUs were obtained by clustering with 97% sequence similarity, with an average of 965 (Table S1). The Shannon index curves revealed that a sufficient number of OTUs were detected and revealed the abundance of microbiota in the samples (Figure S1). The richness and evenness of species in different samples are shown in the rank–abundance curve (Figure S2).

### 3.2. Composition of the Skin Microbiota 

**Figure 1:**
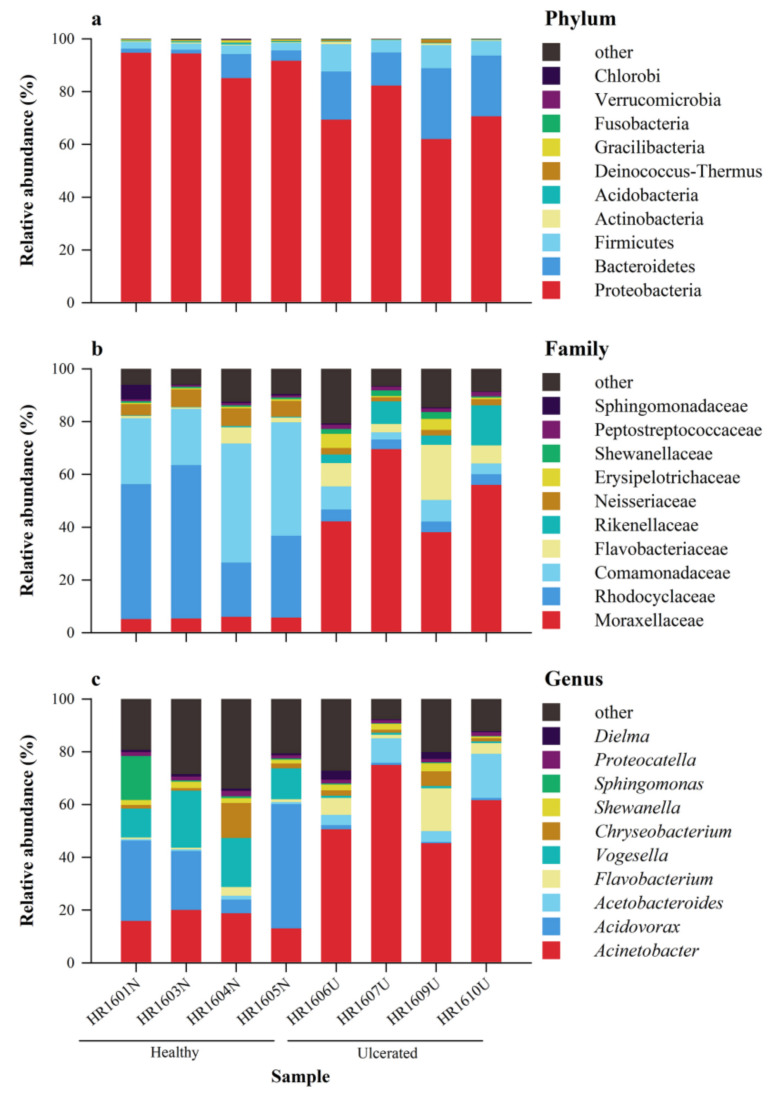
Composition of the skin microbiota of each *Hoplobatrachus* *rugulosus* sample collected to compare healthy and ulcerated skin at the (**a**) phylum, (**b**) family, and (**c**) genus levels. Different colors in the figures indicate different taxa and are labeled on right side of each figure. In each panel, “others” represents the sum of the relative abundance of all other phyla (**a**), families (**b**), and genera (**c**) not listed in the figure.

**Figure 2:**
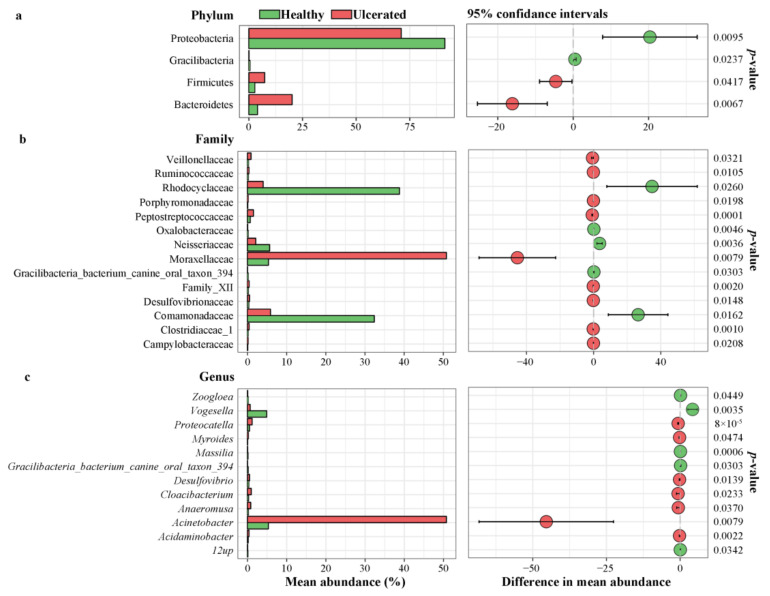
Significant difference analysis for different taxa at the (**a**) phylum, (**b**) families, and (**c**) genus levels between healthy and ulcerated *Hoplobatrachus rugulosus* skin.

Representatives of 34 known phyla, 78 classes, 125 orders, 233 families, and 439 genera of skin microbiota were determined on *H. rugulosus* skin (Table S2). Proteobacteria, Bacteroidetes, and Firmicutes were the highest represented phyla (Figure 1a). Proteobacteria was predominant with a relative abundance of 91.5% ± 4.5% in the HG and 71.1 ± 8.4% in the UG. Bacteroidetes (HG: 4.0% ± 3.6%; UG: 20.1% ± 6.2%) and Firmicutes (HG: 2.7% ± 0.4%; UG: 7.3% ± 2.7%) were the other two major phyla. Meanwhile, the abundance of Proteobacteria and Gracilibacteria were higher in the HG than in the UG, whereas that of Firmicutes and Bacteroidetes were higher in the UG than in the HG (all *p* < 0.05) (Figure 2a). Overall, the dominant phylum observed in both the HG and UG was Proteobacteria, but there was a significant difference in their mean abundance (*p* < 0.01).

The main families were Moraxellaceae, Rhodocyclaceae, Comamonadaceae, Flavobacteriaceae, Rikenellaceae, Neisseriaceae, Erysipelotrichaceae, Shewanellaceae, Peptostreptococcaceae, Sphingomonadaceae, and others (Figure 1b). The most abundant bacterial families in the HG were Rhodocyclaceae (40.2% ± 17.4%), Comamonadaceae (33.7% ± 12.2%), and Neisseriaceae (5.9% ± 1.1%); in the UG, they were Moraxellaceae (51.4% ± 14.3%), Flavobacteriaceae (10.0% ± 7.7%), and Rikenellaceae (7.6% ± 5.6%). The abundance of the 14 families differed between the HG and UG; in particular, the abundance of Rhodocyclaceae, Neisseriaceae, and Comamonadaceae were significantly higher in the HG than in the UG, whereas that of Moraxellaceae was significantly higher in the UG than in the HG (all *p* < 0.05) (Figure 2b). 

The main genera were Acinetobacter, Acidovorax, Acetobacteroides, Flavobacterium, Vogesella, Chryseobacterium, Shewanella, Sphingomonas, Proteocatella, Dielma, and others (Figure 1c). The most abundant bacterial genera in the HG were Acidovorax (26.3% ± 17.5%), Acinetobacter (16.9% ± 3.1%), and Vogesella (15.7% ± 5.3%). Compared with the HG, the UG was rich in Acinetobacter (58.1% ± 13.1%), Acetobacteroides (8.5% ± 6.0%), and Flavobacterium (7.0% ± 6.5%). Furthermore, the abundance of the 12 genera were different between the HG and UG; in particular, the abundance of Vogesella was significantly higher in the HG than in the UG, whereas that of Acinetobacter was significantly higher in the UG than in the HG (all *p* < 0.05) (Figure 2c). 

### 3.3. Differences in Bacterial Operational Taxonomic Units

**Figure 3:**
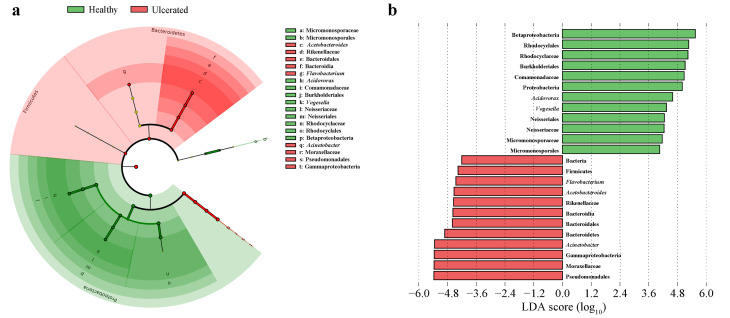
Linear discriminant analysis effect size (LEfSe) of bacterial composition on healthy and ulcerated *Hoplobatrachus* *rugulosus* skin (LDA > 4, *p* < 0.05). (**a**) Taxonomic representation of statistical and biological result reveals consistent differences between healthy and ulcerated *H. rugulosus* skin. Differences are represented using a colored circle; the color of circles represents their respective levels of classification, and the size of circles is proportional to the taxon abundance, representing the phylum, class, order, and family. (**b**) Histogram of the linear discriminant analysis (LDA) scores computed for features differentially abundant between healthy and ulcerated *H. rugulosus* skin. LEfSe scores can be interpreted as the degree of consistent difference in relative abundance of the analyzed bacterial communities between healthy and ulcerated groups.

The taxonomic abundance of the skin bacteria differed between the HG and UG, with the phylum Proteobacteria, class Gammaproteobacteria, order Pseudomonadales, family Moraxellaceae, and genus *Acinetobacter* more abundant in the UG (Figure 2a) than in the HG. In addition, the genus *Acetobacteroides* of the phylum Bacteroidetes, class Bacteroidia, order Bacteroidales, and family Rikenellaceae was dominant in the UG (Figure 3b). The taxonomic groups of the phylum Proteobacteria, class Betaproteobacteria, orders Burkholderiales and Neisseriales, families Comamonadaceae and Neisseriaceae, and genera *Acidovorax* and *Vogesella* were more abundant in the HG than in the UG (Figure 3b).

### 3.4. Alpha and Beta Diversity

Table 1 shows the descriptive statistics of the five alpha diversity indexes of the skin microbiota between the HG and UG. As measured using Good’s coverage index, the sequencing depth averaged 0.995 ± 0.001 for both the HG and UG, suggesting an adequate depth of sequencing. Other alpha diversity indexes showed no significant difference between the HG and UG (all *p* > 0.05).

**Figure 4:**
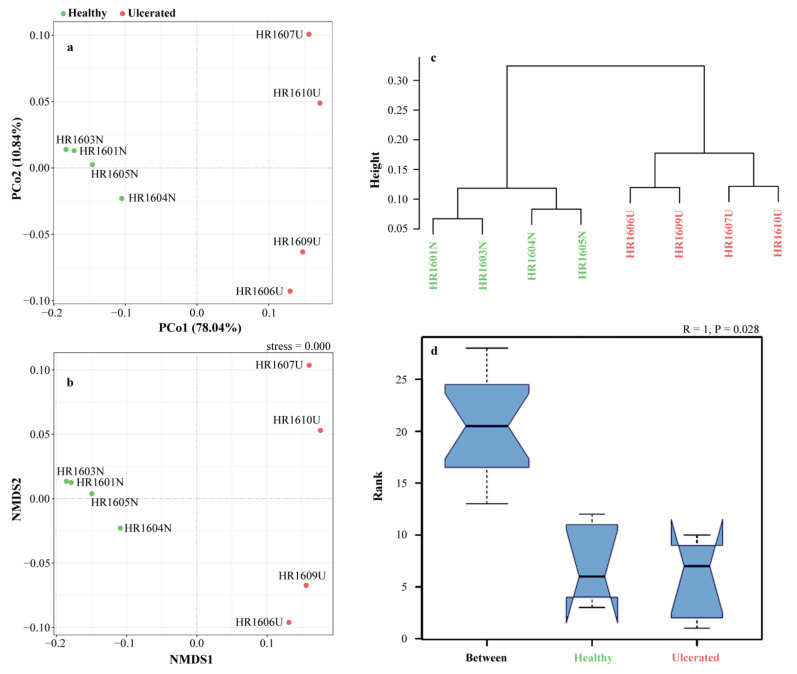
Beta diversity of skin microbiota in healthy and ulcerated groups of *Hoplobatrachus* *rugulosus*. Based on weighted UniFrac distance, (**a**) principal coordinate analysis (PCoA), (**b**) non-metric multidimensional scaling (NMDS), (**c**) unweighted pair-group method with arithmetic means (UPGMA), and (**d**) analysis of similarities (ANOSIM) were performed.

Regarding the beta diversity, in the PCoA diagram, the contribution rate of the PCo1 axis and PCo2 axis were 78.04 and 10.84%, respectively, and the total contribution rate was 88.88%. According to the distribution of sample points in the figure, the two groups of samples are far apart, indicating that the bacteria in the two groups had large structural differences, and the PCo1 axis is the main factor leading to such differences (Figure 4a). The NMDS based on the relative dissimilarities of the samples showed a close aggregation in the HG and UG, respectively, especially in the HG (Figure 4b). The result was validated by the UPGMA (Figure 4c). Additionally, we found that significant differences in the microbial communities were greater between groups than within groups by the ANOSIM test (*p* < 0.05) (Figure 4d).

### 3.5. Differences in Functional Predictions

**Figure 5:**
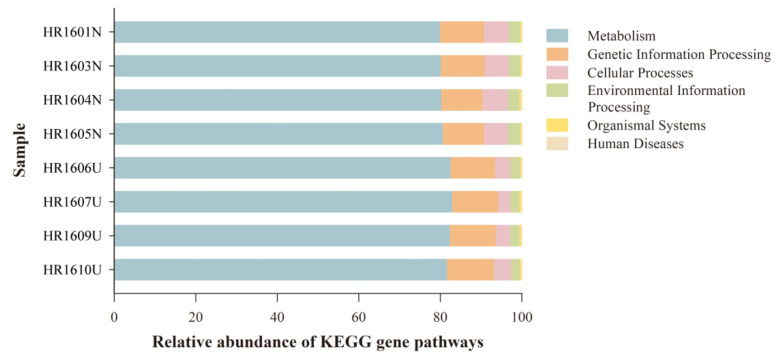
Relative abundance of *Hoplobatrachus rugulosus* skin bacterial taxa at Kyoto Encyclopedia of Genes and Genomes (KEGG) pathways.

The PICRUSt functional prediction showed the abundance of the functional gene related to the KEGG pathways at different levels (1–3). A majority of the KEGG pathways enriched at level 1 were metabolism (81.1% ± 1.2%), genetic information processing (10.9% ± 0.5%), cellular processes (4.8% ± 1.4%), environmental information processing (2.5% ± 0.3%), organismal systems (0.4% ± 0.0%), and human disease (0.2% ± 0.1%) (Figure 5). The results of the Welch’s *t*-test revealed no statistical significance between the HG and UG in the abundance of genes related to the KEGG pathway level 1 (*p* > 0.05). 

**Figure 6:**
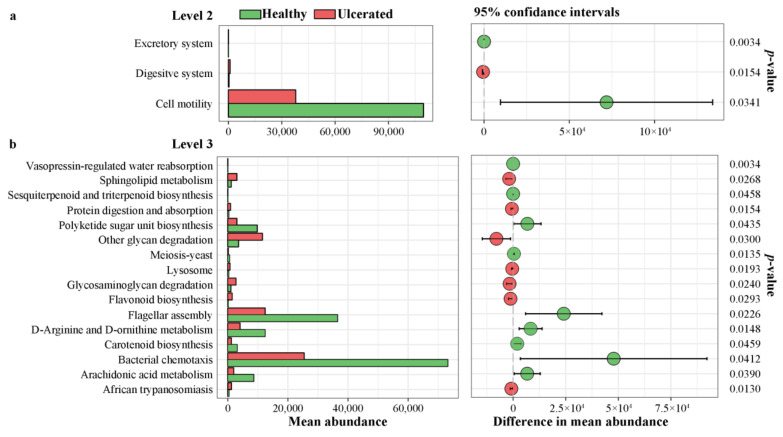
Significant difference analysis of functional classifications of 16s RNA in skin microbiota of *Hoplobatrachus rugulosus* at (**a**) level 2 and (**b**) level 3 between healthy and ulcerated skin.

However, we found that the abundance of the genes related to the KEGG pathways differed significantly between the HG and UG at pathway levels 2 and 3 (Figure 6). In the KEGG pathway level 2, the mean abundance of the genes associated with the excretory system and cell motility was greater in the HG than in the UG, whereas that of the genes associated with the digestive system was smaller in the HG than in the UG (all *p* < 0.05) (Figure 6a). In the KEGG pathway level 3, the mean abundance of the genes associated with the 16 pathways was significantly different between the HG and UG (all *p* < 0.05), especially that of the genes associated with flagellar assembly and bacterial chemotaxis, which were more abundant in the HG than in the UG. In contrast, the genes associated with other glycan degradation were less abundant in the HG than in the UG (Figure 6b).

## 4. Discussion

Disease outbreaks are a major obstacle to the sustainable development of amphibian farming, the most common being skin diseases with ulcerated wounds on the body surface [7]. On one hand, the loss of skin integrity increases the likelihood of pathogenic infection [43,44,45]. Skin damage is very common in captive amphibians, which could be caused by coarse nets in breeding ponds and aggression between members [7]. A further key aspect of amphibian skin biology is skin sloughing [46]. Both result in the breakdown of skin integrity, which may provide a site for pathogen colonization and could contribute to the susceptibility of amphibians to pathogens [47]. Healthy skin also hosts a large number of pathogenic bacteria, which may multiply rapidly and damage the body when the skin is affected [48]. On the other hand, skin microorganisms may participate in a network of microbial–microbial interactions, helping to create a balanced community [49]. Dysregulation of the microbial community structure may lead to increased sensitivity to pathogen infection, especially if the core microbiome composition varies [50]. Recently, there has been growing evidence showing that symbiotic microbes on healthy skin occupy a wide ecological niche and effectively protect individuals from pathogens [51,52]. It was reported that certain symbiotic bacteria (e.g., *Bacillus* sp., *Janthinobacterium* sp., *Pseudomonas* sp., and *Chitinophaga arvensicola*) could cause the production of emergent anti-*Bd* metabolites [17]. Therefore, achieving a complete understanding of the diversity and function of the core microbiota on healthy and ulcerated *H. rugulosus* skin will aid in a better assessment of amphibian health [53].

In this study, we did not find significant differences in the alpha diversity between the healthy and ulcerated skin of *H. rugulosus* individuals, indicating that the skin microbial richness and evenness were similar in the healthy and ulcerated frogs. This result is similar to that reported by Jani and Briggs (2014) [54], who showed that the alpha diversity of the skin microbiota in the Sierra Nevada yellow-legged frog (*Rana sierrae*) did not differ between *Bd-*infected and uninfected individuals. However, our results are not consistent with the observations in the Chinese giant salamander (*Andrias davidianus*) in which skin microbes were more abundant and diverse in healthy individuals than in ulcerated ones. However, there was only one healthy sample in that study [55]. The skin microbial communities of frogs and newts consist of several members of the same families, but the relative abundance of the taxa varies. Thus, we speculated that the discrepancies in the alpha diversity in various studies might be related to differences in the sample sizes and species.

Skin microbial communities can be directly or indirectly affected by environmental factors (e.g., temperature, humidity, and pH) and environmental conditions (e.g., captivity and geographic location) [56,57,58,59], life history [56], and the species studied [60]. Therefore, to eliminate the effect of other variables on skin microbial structural changes, we only considered the skin microbiota from individuals in the same developmental stage that were collected from the same location on the same day. Moreover, all of our samples were obtained from the same dorsal region. The only difference between our samples was the condition of the skin, whether they were healthy or ulcerated, as we aimed to explore the relationship between skin disease and the surface structure of the microbial community in isolation.

The skin microbiota of infected and healthy frogs showed significant differences in beta diversity [20,54]. In our study, the results of the PCoA, NMDS, and UPGMA indicated that there were distinct microbial community structures between the HG and UG, suggesting that the composition of the microbiota on *H. rugulosus* skin was significantly affected by ulceration. Similar results have been reported not only in amphibians but also in fishes as well (e.g., *Salmo salar* and *Sparus aurata*) [44,61].

Proteobacteria, Bacteroidetes, Firmicutes, and Actinobacteria are four of the most important phyla of the skin microbes present in numerous vertebrates [62]. The taxonomic composition at the phylum level showed that the skin microbiome of *H. rugulosus* on both the HG and UG skin is largely comprised of Proteobacteria, followed by Bacteroidetes and Firmicutes. Although our results showed that the dominant phylum of the two groups were the same, the abundance of the microbial community differed greatly. These three phyla were the most common on healthy *H. rugulosus* skin: Proteobacteria was the most prominent (91.3%), followed by Bacteroidetes (3.98%) and Firmicutes (2.72%); however, on ulcerated *H. rugulosus* skin, Proteobacteria, Bacteroidetes, and Firmicutes accounted for 70.98, 20.12, and 7.34%, respectively. These results were different from the findings of Federici et al. [20], who observed similar abundances of bacterial phyla in uninfected and infected Italian stream frogs (*R*. *italica*), except members of the phylum Bacteroidetes.

At the family level, the relative abundance of most of the dominant families differed significantly between the microbiota of the healthy and ulcerated *H. rugulosus* skin. Rhodocyclaceae and Comamonadaceae members were reduced, whereas Moraxellaceae members increased on the ulcerated *H. rugulosus* skin. Moraxellaceae have been associated with high *Bd* loads, and some species of Moraxellaceae were found to have a strong association with *Bd*-induced mortality [63]. It has been reported that skin ulceration can significantly increase or decrease the abundance of certain bacteria in the skin microbiome and cause an imbalance in the microflora [45]. Furthermore, the immune response of the host can be altered at several levels by imbalances or disruptions in the skin microbiota [64]. For example, the *Bd* causes chytridiomycosis, which disturbs the microbial composition of amphibian skin, which then disrupts osmotic regulation in frogs. This fluid imbalance results in organ failure and death [65]. Using microbiomes for a health diagnosis is still an emerging area of research; hence, identifying the core microbiota in individuals is one of the key factors in developing this area [66]. For instance, it appears that an increase in the proportion of the Moraxellaceae may serve as an important threat indicator for *H. rugulosus*.

*Acinetobacter* species are obligate aerobic that are ubiquitous on the skin and also in the soil, water, and rhizosphere of plants [67]. The function of *Acinetobacter* in several previous studies were contradictory. On one hand, it has been widely reported that *Acinetobacter* species cause infections, transmission, and epidemic transmission in hospitals and other healthcare settings, posing a great threat to healthcare systems [68]. On the other hand, they are necessary for the formation of biofilms and serve to structure and protect microbial communities in other biological systems [69,70]. Additionally, *Acinetobacter* isolated from Panamanian amphibians showed anti-*Bd* activity [71]. In this study, the abundance of *Acinetobacter* in the UG was higher than in the HG. The result was consistent with a previous bacterial microbiome evaluation on the dorsal skin of the Ozark hellbender (*Cryptobranchus alleganiensis bishopi*) [72]. Our methodology is currently limited as we did not determine whether the increase in bacteria found on the ulcerated skin is pathogenic or beneficial. Nowadays, many studies on amphibian skin microorganisms mainly use *Bd* as a pathogen to study its inhibitory or enhancing effects on pathogens [73,74,75]; however, the role of other pathogens are not as clear. It is worth noting that *Acinetobacter* was ranked second in proportion to the HG, and this result was consistent with that reported by Becker et al. (2015) [71]. Therefore, future investigations might benefit from incorporating anti-pathogenic factors to determine the impact of skin microbiota on amphibian health.

Probiotics can be used as therapy for preventing diseases, as well as improve growth and survival in many areas, such as human and veterinary medicine, agriculture, aquaculture, and wildlife conservation [76,77,78,79]. Probiotics found on cutaneous microbiota could be introduced to other individuals, and this may potentially reduce the susceptibility of amphibians to skin diseases [80]. For example, Harris et al. [18] described the inhibition of *Bd* by bacteria isolated from the skin of the red-backed salamander (*Plethodon cinereus*) and the eastern four-toed salamander (*Hemidactylium scutatum*). Another study has shown that the bacteria *Pseudomonas reactans*, which was previously shown to be anti-*Bd* in vitro, was established successfully on *P. cinereus*, and the severity of a disease symptom in infected *P. cinereus* decreased [80]. However, it is unfortunate that a probiotic treatment of *Bd*-inhibitory bacteria could not be derived from the infected Panamanian golden frog (*Atelopus zeteki*) that survived *Bd* exposure either by recovering from the infection or by maintaining low *Bd* loads [63]. The clearance or reduction in infection in infected frogs is attributed to the initial composition of skin microorganisms and the production of metabolites by skin microorganisms [63]. By integrating a chemical analysis, an aerobic bacterium (*Janthinobacterium lividum*) was isolated in culture from *P. cinereus* skin and possessed strong antifungal activity as it secreted secondary metabolites which inhibit *Bd* growth [81]. In our study, *Vogesella* was found to be more abundant in the HG and may be a probiotic candidate, according to the report from Jørgensen et al. (2010) [82], which suggested that *Vogesella* was capable of degrading peptidoglycan and had chitinase and lysozyme activities to combat *Bd*. However, the effectiveness of the type of bacteria used in probiotic treatments needs to be further verified by bacterial culture experiments on the inhibitory activity of pathogenic bacteria. Thus, our study serves as a starting framework to further delve into the use of probiotics for amphibians, and our future research will provide more information on this topic.

In this study, the PICRUSt was utilized to predict the potential function from bacterial 16S rRNA sequences, which allowed for the identification of several functional KEGG categories and pathways expressed in *H. rugulosus*. Interestingly, although there were no significant differences in the abundance of the genes related to the KEGG pathway level 1, the predicted results showed that in the KEGG pathway level 2, the HG had a higher gene abundance than the UG, except for the genes associated with the digestive system, which implies that skin ulceration may enhance the function of the digestive system. One reason for this could be that the affected individuals may require a greater amount of nutrients and energy assimilation to accelerate wound healing. Berger et al. (2005) [83] observed the dissolution of cellular cytoplasm in the infected epidermis of the dainty green tree frog (*Ranoidea gracilenta*). Thus, the functional pathways of skin microbes can be affected by ulceration on the surface of the amphibians. Future research may utilize metagenome sequencing of the microbiota on ulcerated *H. rugulosus* skin which may help reveal the underlying functional differences of the microbiota between the healthy and ulcerated skins of frogs.

## 5. Conclusions

Our results revealed that the composition, abundance, and function of microbial communities differed between the healthy and ulcerated *H*. *rugulosus* skin. The abundance of the genes related to the KEGG pathways at levels 2 and 3 in the skin microbiota was greater on the healthy skin than on the ulcerated skin, indicating that skin ulcers could cause functional disorders in *H. rugulosus*. Our results may assist in developing measures to combat the occurrence of diseases in *H. rugulosus*. However, obtaining 16S rRNA gene sequences through the Illumina HiSeq platform has limitations. On the ulcerated skin, 92.7% of the microbial species were unclassified, whereas 89.1% were unclassified on the healthy skin at the species level. As we only compared the differences in the skin microbial structure and the function between the healthy and ulcerated *H. rugulosus* skin, we did not isolate and identify the pathogenic bacteria from the ulcerated *H. rugulosus* skin. Thus, the number of core microbial species that cause skin ulceration is unknown. Additionally, the causes and pathogenesis of skin ulceration are still unclear and need further verification.

## Figures and Tables

**Table 1:** Five alpha diversity indexes of *Hoplobatrachus* *rugulosus* skin microbiota.

Group	Shannon	Simpson	Chao 1	ACE	Good’s Coverage
HG	5.26 ± 0.31 4.90–5.54	0.91 ± 0.02 0.89–0.93	1369.0 ± 178.4 1118.2–1538.3	1416.9 ± 160.7 1181.9–1540.4	0.995 ± 0.001 0.993–0.996
UG	4.76 ± 0.94 3.91–5.63	0.85 ± 0.09 0.76–0.94	1359.5 ± 114.7 1239.4–1498.1	1360.6 ± 111.4 1240.2–1457.3	0.995 ± 0.001 0.994–0.995
Student’s *t* test	*t* = 1.01, *p* = 0.350	*t* = 1.24, *p* = 0.261	*t* = 0.09, *p* = 0.931	*t* = 0.58, *p* = 0.586	*t* = 0.31, *p* = 0.768

## Data Availability

All 16S rRNA gene sequences obtained in this study have been deposited in the NCBI Sequence Read Archive under the BioProject accession number PRJNA766330. Available online: https://www.ncbi.nlm.nih.gov/bioproject/PRJNA766330 (accessed on 7 March 2022).

## Supplementary Materials

The following supporting information can be downloaded at: https://www.mdpi.com/article/10.3390/ani12101241/s1, Figure S1: Shannon index curve of each sample. Different colors of the lines indicate different sample, Figure S2: Rank–abundance distribution curve of each sample. Different colors of the lines indicate different sample, Table S1: Statistics of effective sequences of *Hoplobatrachus rugulosus* skin samples, Table S2: Statistics of different taxonomic levels of *Hoplobatrachus rugulosus* skin microbiota.
