# Supplementary material for: Microbial Diversity of the Chinese Tiger Frog (Hoplobatrachus rugulosus) on Healthy versus Ulcerated Skin
Source: Animals (Basel). 2022 May 12;12(10):1241. doi: 10.3390/ani12101241 (PMC9137582; doi:10.3390/ani12101241)
Supplement: Supplementary file 1 [file animals-12-01241-s001.zip › animals-1659721-supplementary.pdf]

Supplementary Materials

Table S1. Statistics of effective sequences of *H. rugulosus* skin samples.

Sample ID	Total	Taxon	Unclassified	OTUs
HR1601N	73192	70841	0	880
HR1603N	53515	50790	2	1154
HR1604N	74684	71995	2	1035
HR1605N	77015	73757	1	1130
HR1606U	62435	60681	11	870
HR1607U	64563	63119	7	802
HR1609U	61812	60714	5	985
HR1610U	74595	73386	0	866
Total	541811	525283	28	7722
Average	67726	65660	4	965

Table S2. Statistics of different taxonomic levels of *H. rugulosus* skin microbiota.

Sample ID	Domain	Phylum	Class	Order	Family	Genus	Species
HR1601N	2	21	51	84	132	214	81
HR1603N	2	22	56	94	168	251	97
HR1604N	2	19	57	88	148	237	101
HR1605N	2	21	57	95	162	255	97
HR1606U	1	18	52	84	139	233	102
HR1607U	1	16	50	77	135	202	87
HR1609U	1	16	53	84	151	257	96
HR1610U	1	18	58	86	148	235	85
Total	2	34	78	125	233	439	169

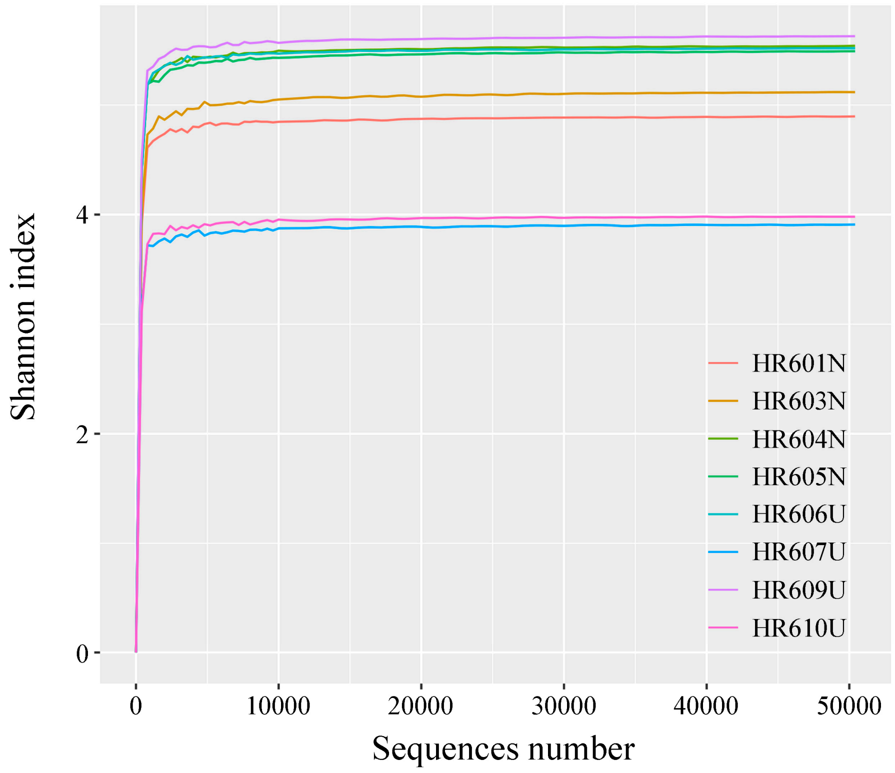

Figure S1. Shannon index curve of each sample. Different colors of the lines indicate different sample.

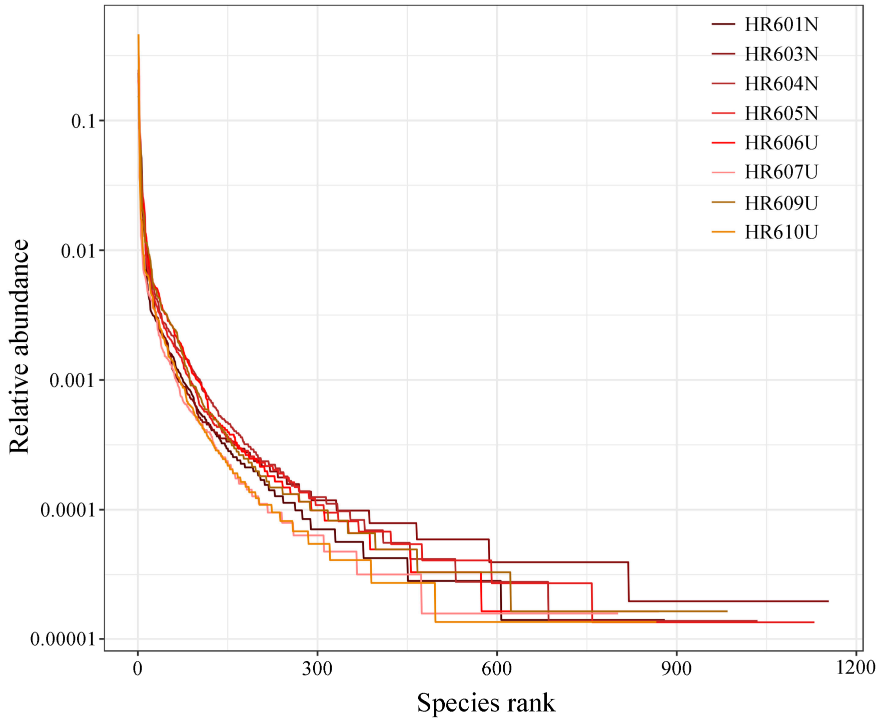

Figure S2. Rank abundance distribution curve of each sample. Different colors of the lines indicate different sample.

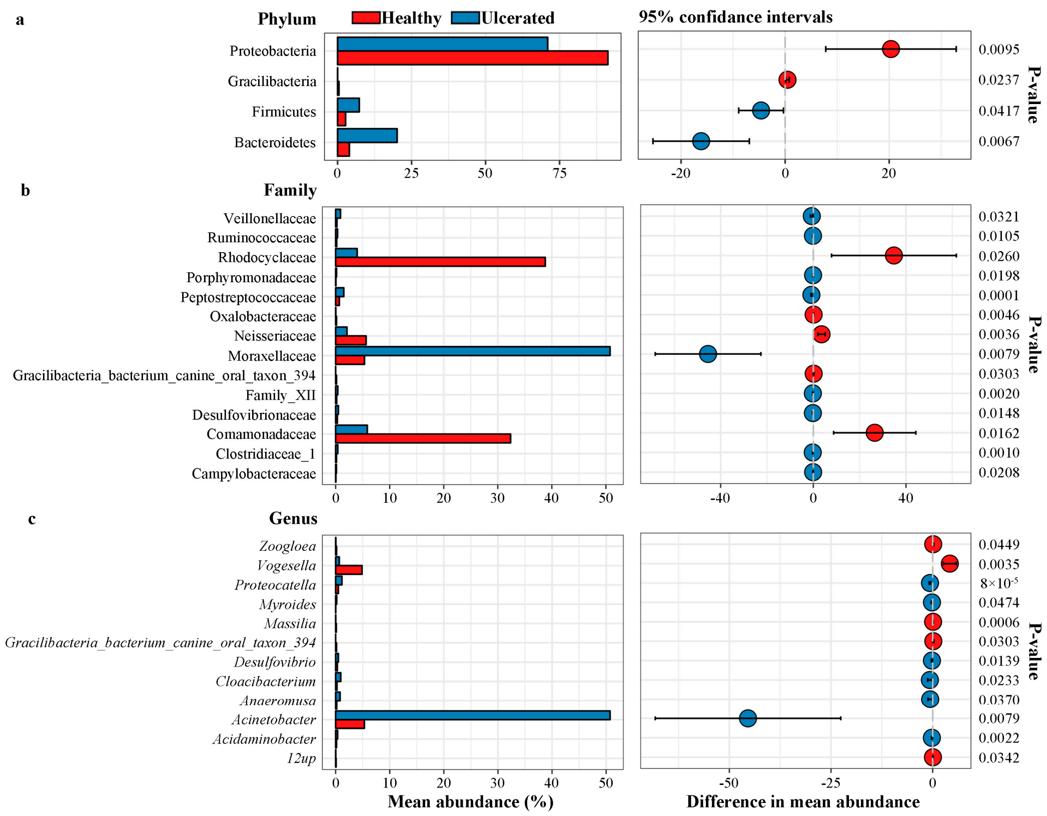

Figure S3. Significant difference analysis for different taxa at the (A) phylum, (B) families, and (C) genus levels between healthy and ulcerated *H. rugulosus* skins.

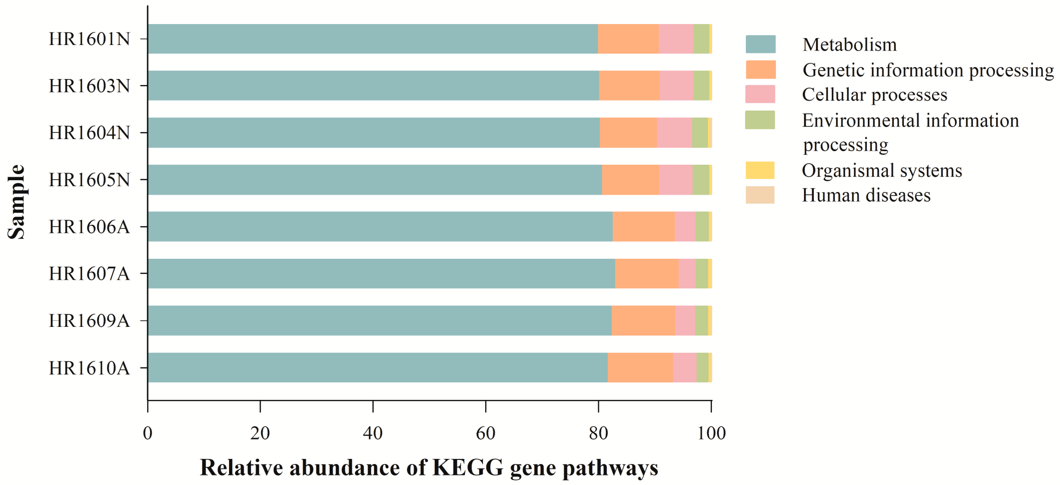

Figure S4. Relative abundance of *Hoplobatrachus rugulosus* skin bacterial taxa at Kyoto Encyclopedia of Genes and Genomes (KEGG) pathways.
